# Hypofractionated radiotherapy with immunochemotherapy for extensive-stage small-cell lung cancer

**DOI:** 10.3389/fimmu.2023.1175960

**Published:** 2023-06-07

**Authors:** Chaoyuan Liu, Liang Zeng, Chao Deng, Wenjuan Jiang, Yapeng Wang, Yiguang Zhou, Li Liu, Sisi Wang, Chunhua Zhou, Zhenhua Qiu, Fanxu Zeng, Fang Wu, Jie Weng, Xianling Liu, Nong Yang, Fang Ma

**Affiliations:** ^1^ Department of Oncology, The Second Xiangya Hospital, Central South University, Changsha, Hunan, China; ^2^ Department of Medical Oncology, Lung Cancer and Gastrointestinal Unit, Hunan Cancer Hospital/The Affiliated Cancer Hospital of Xiangya School of Medicine, Central South University, Changsha, China; ^3^ Department of Oncology, Yueyang Center Hospital, Yueyang, China; ^4^ Department of Oncology, Guilin Hospital of the Second Xiangya Hospital, Central South University, Guilin, China

**Keywords:** extensive-stage small-cell lung cancer, thoracic radiation, immunochemotherapy, safety, progression free survival

## Abstract

**Introduction:**

The combination of a PD-L1 inhibitor plus carboplatin/cisplatin and etoposide (EC/EP) has become a new standard first-line treatment for extensive-stage small-cell lung cancer (ES-SCLC). Combining concurrent palliative hypofractionated radiotherapy of the thorax (HFRT) and immunochemotherapy may have a synergistic effect. In this study, we explored an optimal model of combination radiotherapy with immunochemotherapy as first-line treatment of ES-SCLC.

**Patients and methods:**

In this multicenter single-arm phase 2 trial, patients with ES-SCLC received atezolizumab with EC/EP for two cycles (induction phase), then, those who did not progress received concurrent palliative HFRT and two cycles of atezolizumab with EC/EP (combination phase). Afterward they received atezolizumab every 3 weeks for a maximum of 2 years after study enrolment (maintenance phase). Prophylactic cranial irradiation (PCI) was recommended. The primary endpoints were safety and tolerance; the second endpoints were progression-free survival (PFS).

**Results:**

Forty patients were enrolled, and all had completed palliative HFRT and four cycles of immunochemotherapy. There were seven grade 3 adverse events (3 decreased neutrophil count, 1 anemia, 2 pneumonitis, 1 esoenteritis), two grade 4 adverse events (2 decreased white cell count) and no grade 5 toxicities. The pneumonitis rate was 12.5% (three grade 2 and two grade 3 events). At the median follow-up of 14.2 months (range, 6.8–28.7), the median PFS was 8.6 months (95%CI, 6.1–11.1).

**Conclusion:**

The addition of concurrent hypofractionated thoracic radiotherapy to first-line immunochemotherapy for ES-SCLC was well tolerated and showed promising clinical efficacy. Additional randomized trials are needed to validate benefits.

**Clinical trial registration:**

https://clinicaltrials.gov/ (NCT 04636762).

## Introduction

Small cell lung cancer (SCLC) accounts for 15% of lung cancer cases and is an aggressive cancer characterized by rapid growth, early metastasis, and a poor prognosis (1). Approximately 75% of SCLC patients present with extensive-stage disease at the time of diagnosis, which is classically defined as a disease that cannot be encompassed by a single radiation field (2). Before the era of immunotherapy, the standard first-line therapy for ES-SCLC was platinum-based chemotherapy with etoposide (3); Once complete remission (CR) or partial remission (PR) was achieved after chemotherapy, consolidative thoracic radiation was recommended (4). Despite this standard treatment, the median overall survival (OS) of ES-SCLC is about 8–11 months, which has not changed for about 40 years (5).

Significant progress has been achieved in the treatment of ES-SCLC in recent years. Based on the results of the IMPOWER 133 study and the Caspian study, the PD-L1 inhibitor (Atezolizumab or Durvalumab) with EC/EP has become the new first-line treatment for ES-SCLC (6, 7). However, the results of ES-SCLC remain poor, with a median OS of only approximately 12–13 months (6, 7). There is therefore an urgent need for the development of long-lasting effective treatments for ES-SCLC.

Radiation could induce immunogenic cell death and enhance the antitumor immune response; therefore, synergizing with α-PD-1/PD-L1 inhibitor and can create an abscopal effect (8–10). Previous studies have shown that hypofractionated radiotherapy and immune checkpoint therapy might generate synergistic effects (9, 11). The addition of HFRT to immunochemotherapy may enhance antitumor immunity and improve outcomes (11, 12). The safety and efficacy of combining α-PD-1/PD-L1 inhibitor plus thoracic radiotherapy have been tested in several clinical trials in lung cancer. For example, an international double-blind, placebo-controlled phase III trial PACIFIC showed that adjuvant durvalumab treatment after chemoradiotherapy improved both PFS and OS in patients with stage III NSCLC (13). Some phase 1/2 studies (NCT02621398, NCT02434081, NCT02402920, NCT03585998) also revealed that the α-PD-1/PD-L1 inhibitor plus concurrent chemoradiotherapy combination was tolerable in patients with advanced NSCLC and limited stage SCLC, with promising clinical efficacy (14–16). However, the optimal model for combining immunochemotherapy and radiotherapy remains unknown (17), and there is no report on the safety and efficacy of the combination of concurrent HFRT and immunochemotherapy in ES-SCLC. Therefore, we designed this trial (NCT04636762) to explore a preferred model of combining immunochemotherapy and radiation and to evaluate the safety and efficacy of adding palliative HFRT to standard first-line immunochemotherapy treatment in patients with ES-SCLC. We present a safety profile and a final analysis of PFS.

## Methods

### Patients

The patients were screened at the second Xiangya Hospital, the Hunan Cancer Hospital, and the Yueyang Central Hospital in Hunan province. Inclusion criteria were: 1) adults with histologically confirmed ES-SCLC (the Veterans Administration Lung Study Group staging system) with an Eastern Cooperative Oncology Group (ECOG) performance status score of 0 or 1 (on a 5-point scale, with higher numbers reflecting greater disability); 2) patients with adequate organ function and with no history of previous systemic treatment for ES-SCLC; 3) patients with no disease progression after two cycles of EC/EP with atezolizumab; and 4) patients treated for asymptomatic central nervous system metastases.

The key exclusion criteria were the following: 1) patients with another malignancy that is progressing or requires active treatment; 2) patients with active autoimmune disease or other condition requiring systemic steroids or immunosuppressive agents within the previous 3 months (except for physiological steroid replacement); 3) patients with carcinomatous meningitis; and 4) patients with a history of active Bacillus tuberculosis (TB) or other active infection requiring systemic therapy. Efficacy assessments were performed according to Response Evaluation Criteria in Solid Tumors, version 1.1 (RECIST 1.1).

### Trial design and interventions

The trial was an open, single arm, multicenter, phase 2 trial. The treatment process was divided into three phases: induction, combination, and maintenance phase. The recruited eligible patients received two 21-day cycles of the investigator’s choice of cisplatin (75 mg/m^2^) or carboplatin (area under the curve [AUC] of 5 mg/ml/minute, administered intravenously on day 1 of each cycle) and etoposide (100 mg per square meter of body-surface area, administered intravenously on days 1 through day 3 of each cycle) with concurrent atezolizumab (at a dose of 1200 mg, administered intravenously on day 1 of each cycle) in the induction phase. Cranial irradiation was completed in the induction phase as needed at the discretion of the investigator. If the disease did not progress after the induction phase, the patients were enrolled and the treatment moved to the combination phase. In the combination phase, we added thoracic palliative-hypofractionated radiation therapy to the third cycle of immunochemotherapy. The thoracic radiotherapy protocol involved intensity-modulated radiation therapy based on CT planning, which entailed administering a total dose of 30-45Gy over 10-15 treatment days (equivalent to 14-21 calendar days). The therapy was delivered once daily, with a dose of 3Gy per session. The radiation therapy to the metastatic lesions was permitted at the discretion of the investigators. All radiotherapy procedures were managed by a radiotherapy quality assurance program designed by the Radiation Oncology Department of the Second Xiangya Hospital, Central South University. After the concurrent radiotherapy and the third cycle of immunochemotherapy, the fourth cycle of immunochemotherapy was given without delay. The combination phase was followed by a maintenance phase, in which patients received atezolizumab every 3 weeks for a maximum of 2 years after study enrolment until the appearance of unacceptable toxic effects or disease progression according to RECIST 1.1. During the maintenance phase, prophylactic cranial irradiation (PCI, 25 Gy, 10 fractions) was allowed.

### End points and assessments

The primary endpoints were safety and tolerance. The key secondary endpoint was PFS assessed by the investigator (the time from initial immunochemotherapy to disease progression according to RECIST 1.1 or death from any cause, whichever occurred first) in the population with intention of treatment.

Tumor evaluations were performed at the time of diagnosis, every 6 weeks for the first 18 weeks (starting from day 1 of the first cycle), and every 9 weeks thereafter until the appearance of disease progression according to RECIST 1.1. Adverse events were assessed according to the National Cancer Institute Common Terminology Criteria for Adverse Events, version 4.0. The investigators determined whether adverse events were related to the trial regimen.

### Statistical analysis

Primary and secondary endpoints were evaluated in the intention-to-treat population. The PFS was calculated from the date of immunochemotherapy of the first protocol to the date of progression, death, or the last follow-up, whichever came first. The PFS was estimated by Kaplan–Meier analysis. Summary statistics were calculated along with confidence intervals. The small sample size precluded formal statistical comparison with historical control.

## Results

### Patients

**Figure 1:**
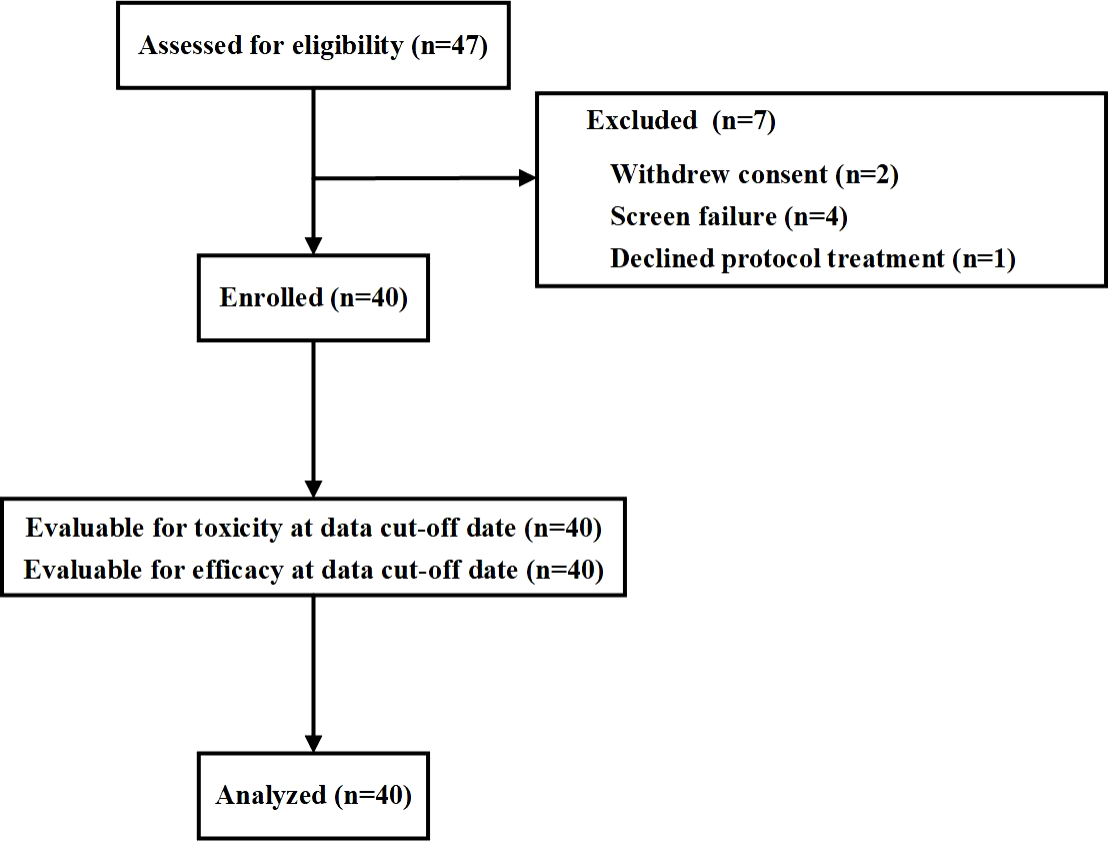
Flowchart showing study eligibility and analysis.

Between 6 June 2020 and 31 November 2021, a total of 40 patients were enrolled at three sites in China. The eligibility and analysis flow chart is shown in **Figure 1**.

The demographics of the baseline patient and the characteristics of the disease are shown in **Table 1**. Most of the participants were males (97.5%), current or former smokers (92.5%), and the median age was 58 years old (range, 47 to 75). Most had metastatic disease with an ECOG score of 1.

**Table 1:** Baseline characteristics of all enrolled patients (Intention-to-Treat Population).

Characteristics	no. (%)
Mean age (min, max) -- years	58 (47,75)
Age group, years	
<65	27 (67.5%)
≥65	13 (32.5%)
Sex	
female	1 (2.5%)
male	39 (97.5%)
Smoking status	
Never smoked	3 (7.5%)
Current smoker	32 (80%)
Former smoker	5 (12.5%)
Disease stage	
III	9 (22.5%)
IV	31 (77.5%)
ECOG score	
0	7 (17.5%)
1	33 (82.5%)
Brain metastasis at enrollment	3 (7.5%)
Liver metastases at enrollment	6 (15%)

### Treatment

The treatment exposure in the 40 enrolled patients is shown in **Table 2**. The median number of atezolizumab doses received was 9 (range, 4 to 29). Eight (20%) patients received 12 or more doses of atezolizumab. All patients received four cycles of platinum–etoposide, half received cisplatin, and the remainder received carboplatin. Six (15%) patients received PCI in the maintenance phase.

**Table 2:** Treatment exposure (safety population).

Atezolizumab (N=40)	
Median number of atezolizumab doses, median (min, max)	9 (4, 29)
Patients receiving 12 or more atezolizumab doses	8 (20%)
Platinum	
Cisplatin	20 (50%)
Carboplatin	20 (50%)
Patients receiving PCI	6 (15%)

PCI, Prophylactic cranial irradiation.

### Safety

The safety of all 40 patients was evaluated. Adverse events related to any component of the trial regimen occurred in 39 patients (97.5%). The most common grade 3 or 4 adverse events related to the trial regimen were a decrease in white blood cell count (**Table 3**). No death related to the trial regimen occurred. Thyroid dysfunction was the most common immune-related adverse event; it appeared in 10 patients (25%), with grade 1–2 hypothyroidism. Treatment-associated pneumonitis occurred in 5 patients (12.5%); only two of them were grade 3 (5%); the remainder were grades 1 or 2. Of these immune-related adverse events, only 3 patients discontinued maintenance treatment with atezolizumab due to grade 3 esoenteritis (1 patient) or grade 3 pneumonitis (2 patients, who recovered after active treatment with corticosteroids).

**Table 3:** The incidence of Adverse events of any cause (safety population).

Adverse events	Any Grade-- (no,(%))	Grade 3-- (no,(%))	Grade 4-- (no,(%))
Any event	39 (97.5%)	7 (17.5%)	2 (5%)
Any event leading to discontinuation	3 (7.5%)	3 (7.5%)	0
Decreased white cell count	21 (52.5%)	3 (7.5%)	2 (5%)
Decreased platelet count	4 (10%)	0	0
Anemia	25 (62.5%)	1 (2.5%)	0
Alopecia	14 (35%)	0	0
Nausea	12 (30%)	0	0
Fatigue	8 (20%)	0	0
Decreased appetite	9 (22.5%)	0	0
Vomiting	5 (12.5%)	0	0
Constipation	4 (10%)	0	0
Diarrhea	3 (7.5%)	0	0
Hypo-albuminemia	2 (5%)	0	0
Pneumonitis	5 (12.5%)	2 (5%)	0
Thyroid dysfunction	10 (25%)	0	0
Myocarditis	1 (2.5%)	0	0
Esoenteritis	1 (2.5%)	1 (2.5%)	0
Esophagitis	12 (30%)	0	0

Multiple occurrences of the same adverse event in one patient were counted once at the highest grade for the preferred term. The incidence of treatment‑related adverse events associated with any component of the trial regimen is shown. no. (%): "no" means the number of patients who have the according adverse events, and "%" means the incidence of the according adverse events.

### Progression-free survival analysis

**Figure 2:**
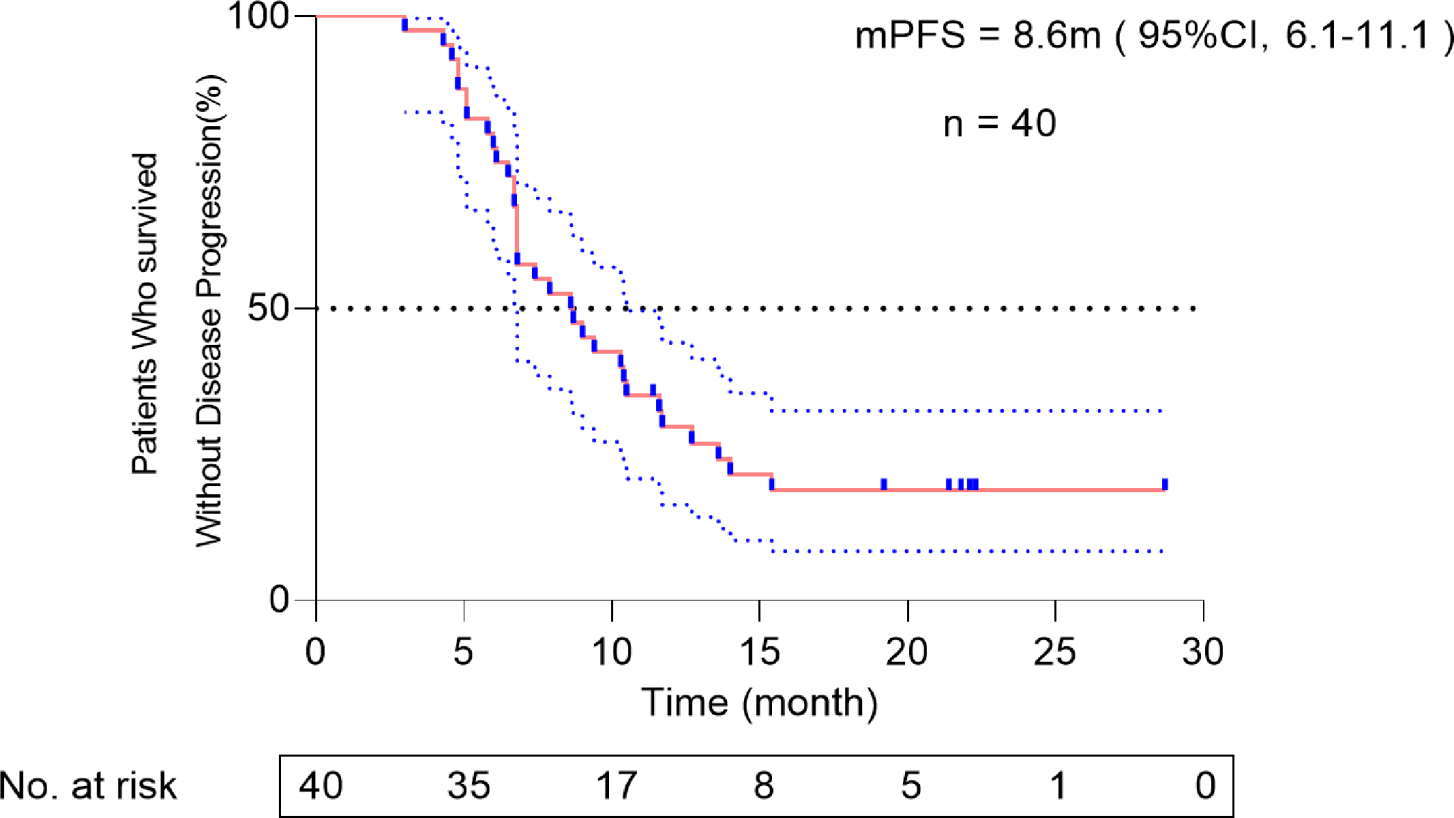
Kaplan–Meier plots of progression-free survival. The blue dot lines represent 95% CI of PFS.

The data cutoff date was 26 October 2022. The median follow-up time was 14.2 months (range, 6.8–28.7), the median PFS was 8.6 months (95%CI, 6.1–11.1) (**Figure 2**). The PFS rate at 12 months was 27.5%. PFS according to baseline characteristics is shown in **Table 4**.

**Table 4:** PFS according to baseline characteristics.

	No. of Patients (%)	Median PFS (months)	HR (95%CI)
Age group			
<65 years	27 (67.5%)	7.90	0.98 (0.94-1.03)
≥65 years	13 (32.5%)	10.30	
Sex			
Female	1 (2.5%)	Undefined	Undefined
Male	39 (97.5%)	8.60	
Smoking status			
Never smoked	3 (7.5%)	11.60	1.61 (0.80-3.24)
Current smoker	32 (80%)	6.80	
Former smoker	5 (12.5%)	11.70	
Disease stage			
III	9 (22.5%)	9.40	1.596 (0.66-3.89)
IV	31 (77.5%)	7.90	
ECOG score			
0	7 (17.5%)	7.40	1.041 (0.40-2.71)
1	33 (82.5%)	8.70	
Brain metastasis at enrollment			
YES	3 (7.5%)	6.67	2.66 (0.28-24.97)
NO	37 (92.5%)	8.63	
Liver metastases at enrollment			
YES	6 (15%)	6.70	1.69 (0.53-5.42)
NO	34 (85%)	9.37	

### Confirmed objective response rate

**Figure 3:**
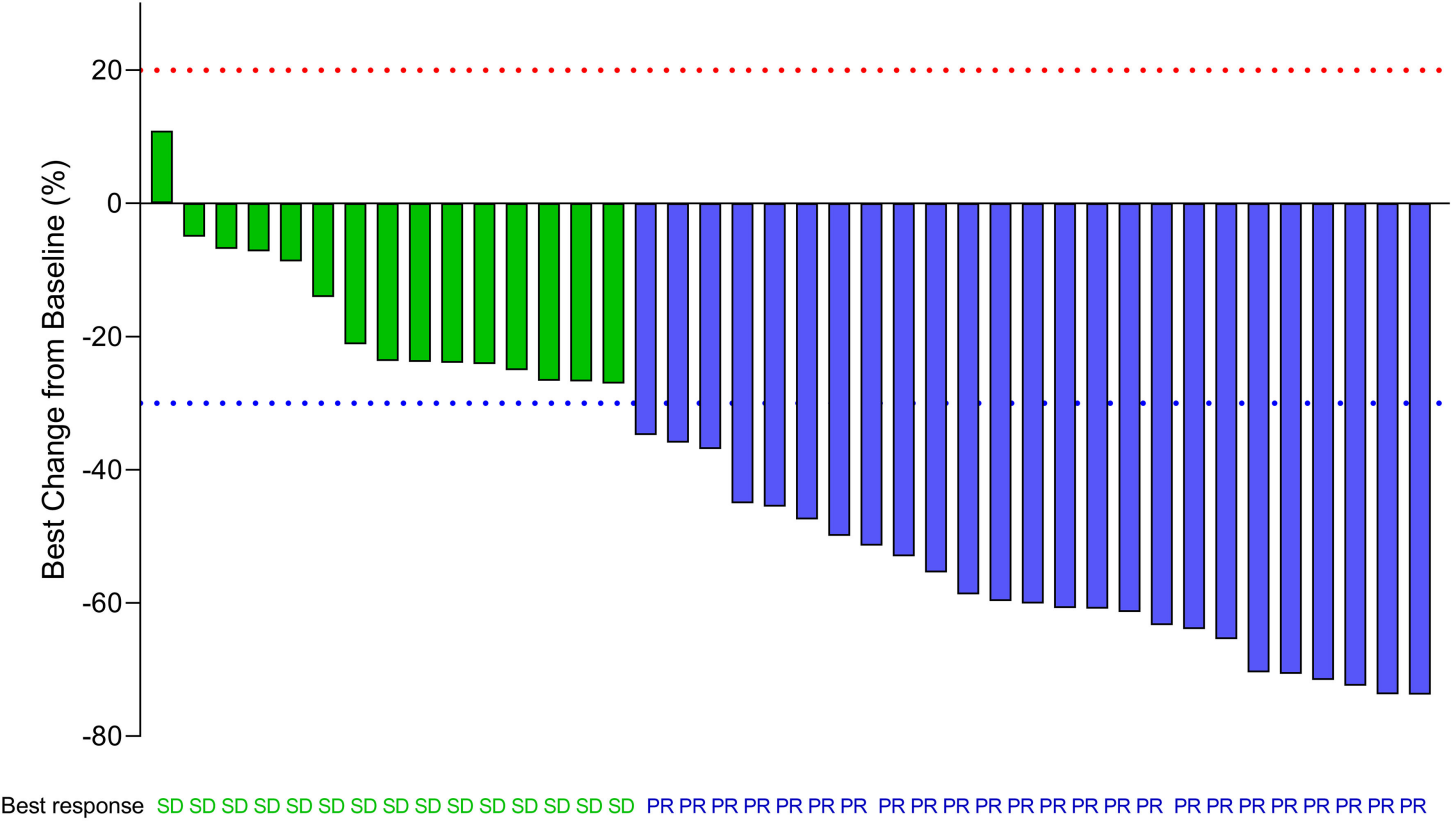
Waterfall map showing the best response of enrolled patients. The waterfall plots display an individual patient’s best response data expressed as the percent change in the sum of the longest diameter of target lesions as measured at baseline and the best response during the whole treatment period. (SD, PR) is indicated by the color of the data bar. SD, stable disease; PR, partial response. The red dot line represents 20% increase in the sum of the longest diameter of target lesions than baseline according to RECIST 1.1. The green dot line represents 30% decrease in the sum of the longest diameter of target lesions than baseline according to RECIST 1.1.

The investigator-assessed confirmed objective response rates (from the start of the screening to the time of enrollment) are shown in **Table 5**. In total, 32 (32/44,72.7%) patients achieved a partial response (PR) and 40 (40/44,90.9%) patients achieved disease control. A waterfall map of the best response of the enrolled patients is shown in **Figure 3**. In total, the lesions of 39 (39/40,97.5%) patients decreased, and 25 (25/40,62.5%) patients achieved PR.

**Table 5:** Summary of tumor responses.

Best response after first two cycles of immunochemotherapy	
CR	0
PR	32
SD	8
PD	4

CR, complete remission; PR, partial remission; SD, stable disease; PD, progressive disease.

## Discussion

Radiotherapy plays an important role in ES-SCLC. A large phase 3 randomized controlled trial CREST showed that the addition of thoracic radiotherapy prolonged progression-free survival at 6 months (24% vs. 7%, P=0.001) and the 2-year OS (13% vs. 3%, P=0.004) significantly. Thoracic radiotherapy was thus recommended for all patients with ES-SCLC who respond to chemotherapy (18). The NCCN guidelines also recommend thoracic radiotherapy if ES-SCLC achieves CR or PR after chemotherapy. However, in the era of immunotherapy, the optimal timing, radiation schedule, safety, and efficacy of thoracic radiotherapy in ES-SCLC have yet to be evaluated. In limited-stage SCLC, concurrent chemoradiotherapy is more effective than sequential chemoradiotherapy (19), and thoracic radiation should be initiated in the first or second cycle of chemotherapy (20–22). Thus, earlier radiation may be beneficial for SCLC. Furthermore, some studies have shown that compared to post-immunotherapy radiation, pre-immunotherapy or concurrent radiation could induce more potent abscopal responses (23, 24). Our study added hypofractionated thoracic radiation after two cycles of immunochemotherapy in ES-SCLC patients who responded. There are two similar ongoing trials registered on the ClinicalTrials website. These trials were designed to explore the safety and efficacy of concurrent radiation and immunochemotherapy in recurrent ES-SCLC or in ES-SCLC refractory to initial platinum-based chemotherapy (NCT03262454, NCT04562337), and did not investigate the first-line treatment evaluated in our study. To our knowledge, this study is the first report of a prospective trial evaluating the safety and efficacy of concurrent thoracic palliative-hypofractionated radiation plus immunochemotherapy in patients with ES-SCLC.

The addition of concurrent thoracic palliative-hypofractionated radiotherapy after two cycles of immunochemotherapy is the key difference between the regimen adopted in the present study and the atezolizumab regimens in the IMPOWER 133 trial. The follow-up time was about 14 months (ours vs. IMPOWER 133, 14.2 months vs. 13.9 months). The median number of atezolizumab doses used was comparable between the present study and the IMPOWER 133 study: 9 (range, 4 to 29) vs. 7 (range, 1 to 30), respectively. Adverse events related to any component of the trial regimen occurred in 97.5% of the patients (in IMPOWER 133, 94.9%). The most common all-grade adverse events were anemia and decreased white blood cell count. The shared grade 3 or 4 adverse events related to the trial regimen were a reduced white blood cell count. Unlike IMPOWER133, there was no Grade 5 adverse event in the present study. The most immune-related adverse event in both studies was hypothyroidism. Some studies reported increased pneumonitis when combining immunochemotherapy and thoracic radiation (9, 25). All-grade and grade 3-5 pneumonitis was higher in the present study compared to the IMPOWER 133 study, 12.5% (5/40) vs. 4% (8/198) and 5% (2/40) vs. 2.5% (5/198), respectively. Nevertheless, there was no grade 5 pneumonitis in our study. The incidence of all-grade or grade 3-5 pneumonitis in our study is comparable to those reports that combined immunochemotherapy and thoracic radiotherapy in NSCLC (25, 26). In SCLC, a Phase I/II Trial reported the incidence of all-grade pneumonitis of the regimen of pembrolizumab and concurrent chemoradiation therapy for limited-stage small cell lung cancer was 15% (6/40), grade 1-2 and grade 3 effects were half-to-half (15), which was more or less similar to those observed in our study. In the present study, although more patients experienced pneumonitis than those in the IMPOWER 133 trial, all patients recovered after active treatment with corticosteroids. In summary, the addition of thoracic radiation to immunotherapy leads to a higher incidence of pneumonitis, but it is manageable. Due to the short follow-up time in the current study, it is not possible to assess long-term toxic effects such as pulmonary fibrosis and esophageal stricture or fistula. In summary, first-line concurrent thoracic palliative-hypofractionated radiation plus immunochemotherapy for ES-SCLC has an acceptable safety profile, at least in the short term.

The PFS in the present study is approximately 3.4 months longer than that of IMPOWER 133(8.6 months (95%CI, 6.1–11.1)) vs. 5.2 m (95% CI, 4.4–5.6)). The PFS rate at 12 months is 27.5%. An abstract in the 2021 ESMO congress (Abstract NO.#2568) reported a retrospective study that showed a significant improvement in PFS for patients with ES-SCLC undergoing atezolizumab and consolidating thoracic radiotherapy, which is consistent with our study. In the retrospective study mentioned above, consolidating thoracic radiation therapy was performed during the atezolizumab maintenance phase; the detailed radiation dose and fractionation schedules remained unknown, which differed from our study. The previous study did not report any benefit in overall survival (OS) for patients undergoing the combination of atezolizumab and consolidating thoracic radiotherapy. Whether the PFS benefit revealed in our study could convert into an OS benefit requires further follow-up.

Unlike in the IMPOWER 133 trial, the PFS in patients with treated brain or liver metastases was shorter than in those without brain or liver metastases in our study. However, no conclusions can be drawn due to the small number of patients with brain or liver metastases enrolled in the trial. Similar to the IMPOWER 133 trial, we also noticed that older patients had a longer PFS than younger patients. Further analyses are needed to explore the potential mechanisms.

This phase II trial has its limitations. First, the sample size was small. Second, the follow-up time was relatively short and we did not have the final analysis of OS. Third, this study was a single-arm and open trial, some inevitable biases included selection bias, differential and non-differential reporting bias, and confounding effects. However, these limitations could not obscure its contributions to exploring an optimal model of first line treatment for ES-SCLC.

In summary, this open, single-arm, multicenter, phase 2 trial showed that the addition of concurrent thoracic palliative-hypofractionated radiation therapy to first-line standard immunochemotherapy resulted in significantly longer PFS than immunochemotherapy, with a manageable safety profile. Our findings laid a foundation for further randomized investigations.

## Data availability statement

The raw data supporting the conclusions of this article will be made available by the authors, without undue reservation.

## Ethics statement

The studies involving human participants were reviewed and approved by the Declaration of Helsinki and Good Clinical Guidelines and ethics committee and the institutional review board of the Second Xiangya Hospital of Central South University (2020–089). All patients provided their written informed consent to participate in this study.

## Author contributions

Main contribution: XL: Conceptualization, Methodology. NY: Conceptualization, Methodology. FM: Conceptualization, Methodology. CL: Data curation, Writing- Original draft preparation. LZ: Data curation, Writing- Original draft preparation. CD: Investigation, Supervision. WJ: Investigation, Writing- Reviewing and Editing. YZ, Data curation. YW: Formal analysis, Investigation. LL: Investigation. SW: Investigation, Validation. CZ: Investigation. ZQ: Investigation. FZ: Formal analysis, Investigation. FW: Investigation, Supervision. JW: Investigation, Supervision. All authors contributed to the article and approved the submitted version.
